# Bilateral Lupus Chorioretinopathy in a Patient With Active Systemic Lupus Erythematosus

**DOI:** 10.7759/cureus.30081

**Published:** 2022-10-08

**Authors:** Sharoon David, Sarah O Davidson, Ruben Grigorian

**Affiliations:** 1 Ophthalmology, Eye Associates of Northeast Louisiana, West Monroe, USA

**Keywords:** serous retinal detachment, ocular manifestations, systemic lupus erythematosus, lupus, retinopathy, choroidopathy, chorioretinopathy

## Abstract

Ocular involvement is commonly seen in systemic lupus erythematosus (SLE). However, chorioretinopathy is an easily missed ocular manifestation of SLE. Early recognition and a multidisciplinary treatment approach can play a key role in reducing the ocular and systemic morbidity seen with this condition. This case report describes a patient with active SLE who presented with bilateral lupus chorioretinopathy. The patient demonstrated a significant improvement in ocular symptoms once the systemic disease was controlled.

## Introduction

Systemic lupus erythematosus (SLE) is a clinically heterogeneous inflammatory disorder that is autoimmune in origin and is characterized by the development of autoantibodies to nuclear antigens. SLE has a wide range of clinical manifestations and displays a variable clinical course. It can affect any organ system of the body but most commonly presents with the involvement of skin (rash), musculoskeletal system (arthritis), and fatigue [1-3]. Ocular involvement is reported to occur in about one-third of the patients. The most common ocular manifestation of SLE is keratoconjunctivitis sicca. Other ophthalmologic manifestations such as episcleritis, scleritis, keratitis, iridocyclitis, retinopathy, choroidopathy, and optic neuropathy have also been described. Lupus retinopathy can occur in 3-29% of patients with active disease, making it one of the most common presentations of active disease. However, lupus choroidopathy is an extremely rare manifestation and has been described in only a few patients [3-5].

We present the case of a 30-year-old female who developed bilateral lupus chorioretinopathy 13 years after the diagnosis of SLE. The chorioretinopathy resolved after appropriate management of the systemic disease.

## Case presentation

A 30-year-old African American woman with a history of SLE presented to our clinic complaining of decreased vision in both eyes for two weeks. She also noticed a “large black dot centrally” in the left eye. The patient reported having problems seeing through her right eye since birth. Her past medical history was significant for SLE, end-stage renal disease, hypertension, osteopenia, and cardiomyopathy. She had no history of diabetes. The patient had been diagnosed with SLE 13 years ago and treated with hydroxychloroquine and methotrexate but had been noncompliant with the treatment. However, at the time of the presentation, she reported that she was taking hydroxychloroquine and prednisone. Her other medications included hydrochlorothiazide, carvedilol, and hydrocodone. She had a history of two cesarean sections and was not pregnant at the time.

**Figure 1:**
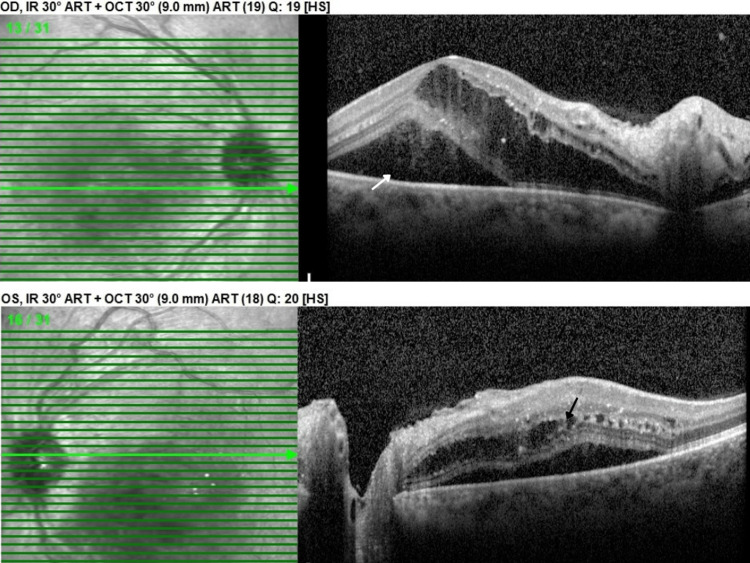
Optical coherence tomography OU - initial visit: macular edema with lipid exudates, subretinal fluid (white arrow), and intraretinal fluid (black arrow)

On presentation, she had a visual acuity of 20/400 in the right eye and finger count in the left eye. Intraocular pressure was 18 mmHg in the right eye and 16 mmHg in the left eye. A biomicroscopic examination of the anterior segment revealed no abnormalities in either eye. Examination of the vitreous was also unremarkable. Examination of the posterior pole revealed multiple intraretinal hemorrhages and cotton wool spots in all four quadrants of both eyes. In addition, serous retinal detachment was noted bilaterally. Optical coherence tomography (OCT) showed the presence of intraretinal and subretinal fluid in both eyes (Figure 1).

**Figure 2:**
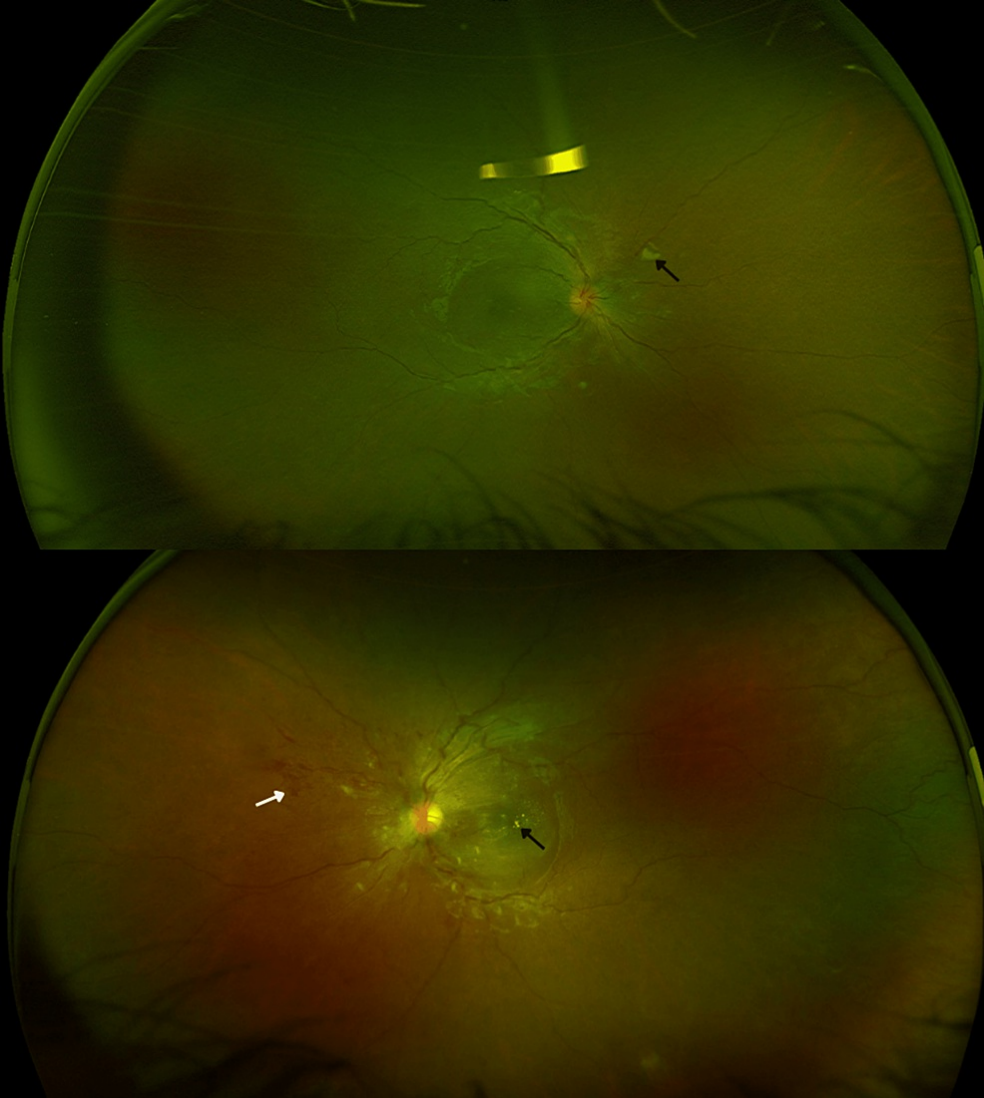
Ultra-wide field fundus photography OU - initial visit: intraretinal deposits (black arrows), intraretinal and subretinal hemorrhages (white arrow), and tortuous veins

**Figure 3:**
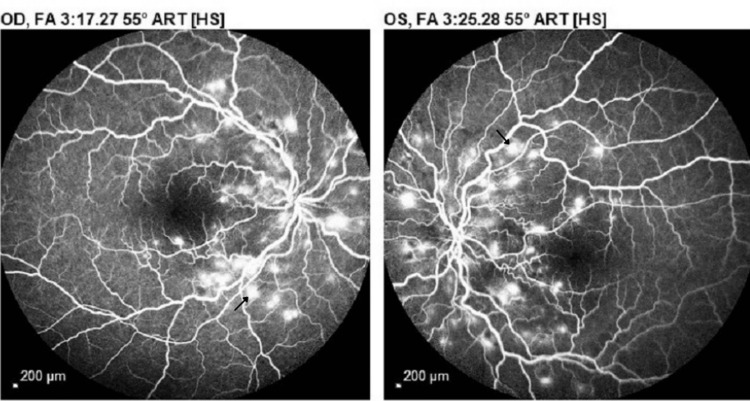
Fluorescein angiography OU - initial visit: multiple hypofluorescent spots consistent with intraretinal hemorrhages and hyperfluorescent areas (black arrows) consistent with dye leakage during the full venous phase

Ultra-wide field fundus photography revealed multiple intraretinal deposits, intraretinal and subretinal hemorrhages, and tortuosity of veins. (Figure 2). Fluorescein angiography demonstrated multiple areas of hypofluorescence consistent with intraretinal hemorrhages and multiple hyperfluorescent areas consistent with leakage bilaterally (Figure 3).

Based on the examination findings, the patient was diagnosed with bilateral lupus chorioretinopathy and was immediately referred to a rheumatologist for the management of SLE. The patient defaulted from follow-up but then returned to our clinic after seven months. She reported that she was following up with her primary care physician (PCP) for the management of SLE and was taking her medications regularly. Her medications included hydroxychloroquine, labetalol, clonidine, doxazosin, furosemide, amlodipine, lisinopril, hydralazine, famotidine, hydrocodone, temazepam, vitamin B12, and thiamine. She stated that her vision was still blurry; however, she did not notice any “black spots” in her vision now. Her visual acuity at this visit was 20/400 in the right eye and 20/200 in the left eye. The intraocular pressure was 15 mmHg in the right eye and 14 mmHg in the left eye. Examination of the anterior segment was normal. The examination of the posterior pole revealed few intraretinal hemorrhages with white-yellowish deposits in the foveal area and no evidence of serous retinal detachment in either eye.

**Figure 4:**
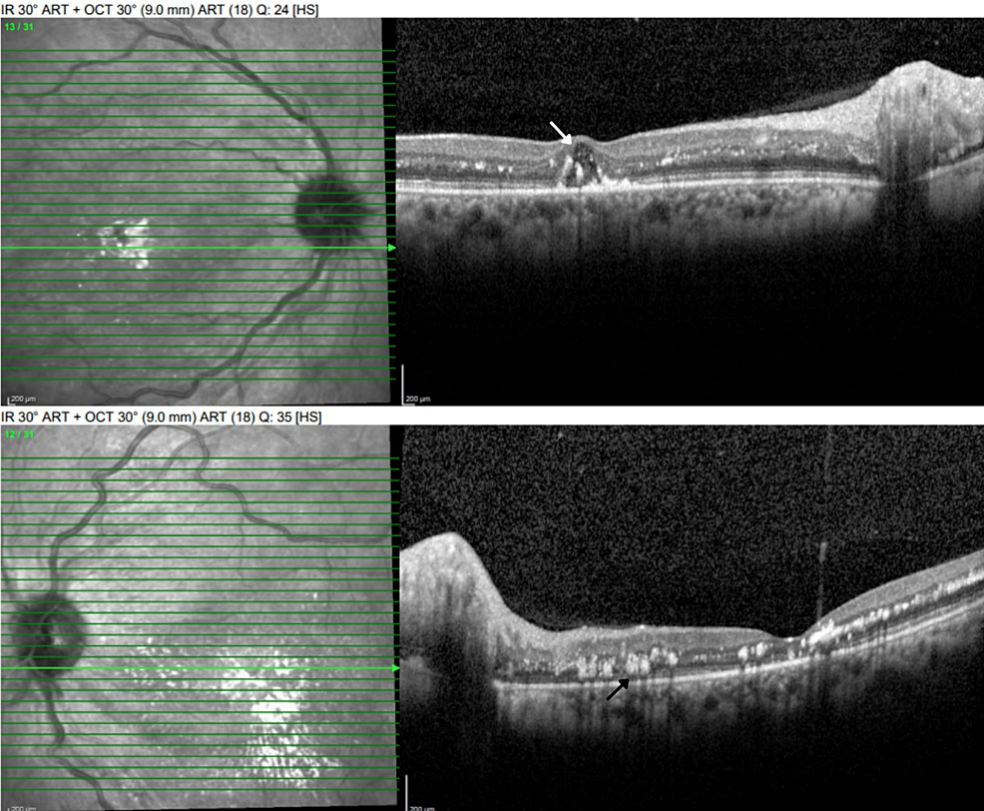
Optical coherence tomography OU - follow-up visit: multiple intraretinal deposits (black arrow) and thinning of the outer retina (absence of ellipsoid zone). Some cystic changes were also noted in the right eye (white arrow)

OCT examination revealed multiple intraretinal deposits with cystic changes in the fovea and thinning of the outer retina (absence of ellipsoid zone) in both eyes. The right eye also had some cystic changes in the fovea (Figure 4).

**Figure 5:**
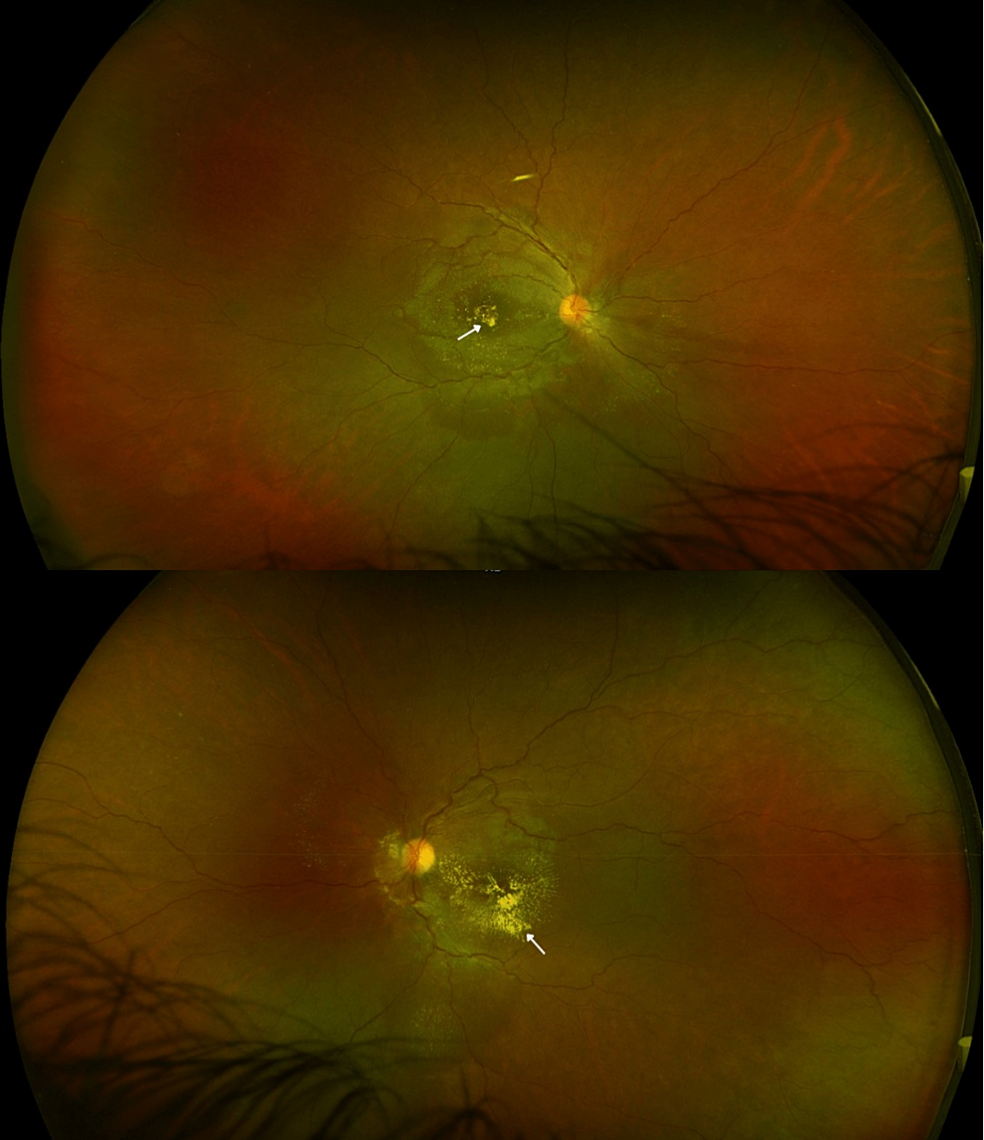
Ultra-wide field fundus photography OU - follow-up visit: multiple intraretinal deposits (white arrows), parafoveal/perifoveal depigmentation with few intraretinal hemorrhages, and tortuosity of veins bilaterally

**Figure 6:**
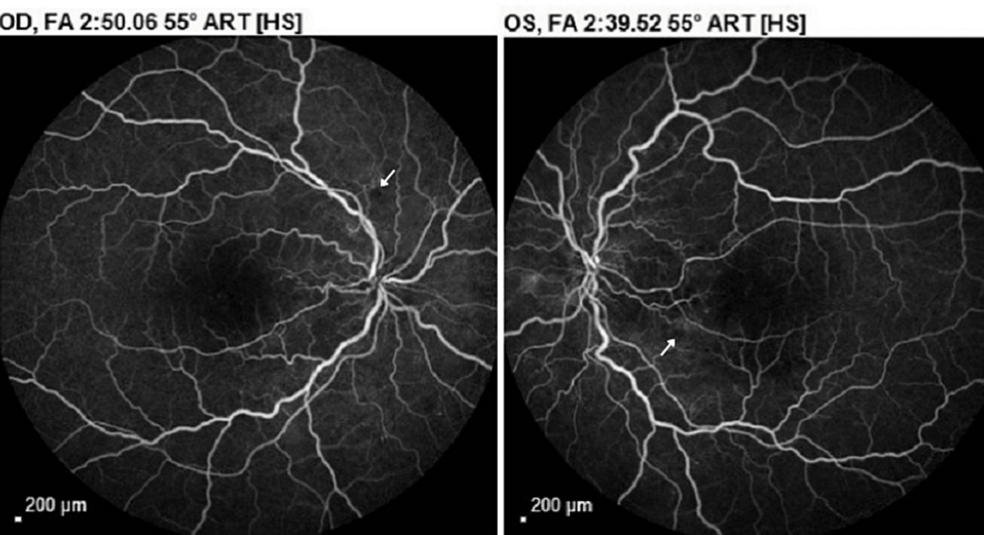
Fluorescein angiography OU - follow-up visit: enlarged foveal avascular zone (FAZ) and multiple hypofluorescent areas consistent with intraretinal hemorrhages (white arrows). No evidence of leakage was present

Ultra-wide field fundus photography revealed multiple intraretinal deposits, parafoveal and perifoveal depigmentation, few intraretinal hemorrhages, and tortuosity of veins bilaterally (Figure 5). Fluorescein angiography demonstrated hypofluorescent spots, consistent with intraretinal hemorrhages, and enlarged irregular foveal avascular zone (FAZ) in both eyes without any evidence of leakage or neovascularization (Figure 6). The patient was advised to continue follow-up with her PCP and to follow up for a retina evaluation in six months.

## Discussion

SLE is an autoimmune inflammatory disorder that has a variable clinical course and affects multiple organ systems of the body. It is almost 10 times more common in females as compared to males and usually has a predilection for women of childbearing age. Asians and Afro-Caribbeans are most commonly affected [1-3]. The precise pathological mechanism involved in the development of SLE is still not fully understood; however, the majority of patients have increased levels of autoantibodies directed against nuclear components, such as DNA, nucleosomes, and histones [1,6,7]. The most common pattern of presentation is a mixture of constitutional symptoms, such as fever, fatigue, and weight loss, along with skin, musculoskeletal, and hematologic involvement. However, some patients may have predominantly renal, hematologic, or central nervous system manifestations [3,8].

Ocular symptoms may also be seen in patients with SLE and can indicate the presence of active systemic lupus. SLE can involve several parts of the eye. Keratoconjunctivitis sicca is considered to be the most common ocular manifestation, with the majority of patients presenting with at least one dry eye symptom. Most patients with SLE develop secondary Sjögren’s syndrome. Corneal epitheliopathy, ulceration, scarring, and filamentary scarring may occur secondary to keratoconjunctivitis sicca. Orbit and optic nerve involvement have also been reported but are extremely rare [4,5,9].

Lupus retinopathy is the most common posterior segment finding seen in patients with SLE. It is a vision-threatening complication and may occur in up to 29% of patients with active systemic disease. Microangiopathy, which is identical to hypertensive and diabetic retinopathy, is the most common pattern of retinal involvement observed in SLE. Cotton wool spots and intraretinal hemorrhages are the earliest findings. Immune-complex deposition in the vessel walls and microemboli are involved in the pathogenesis of this condition. Lupus retinopathy is associated with a poor visual prognosis with the visual loss occurring in almost 80% of patients and neovascularization in around 40% of cases [10,11].

Involvement of the choroid is an extremely rare manifestation of SLE that can result in loss of vision. Approximately 40 cases of choroidopathy occurring in association with SLE have so far been described [12]. The first evidence for choroidal involvement in SLE patients was provided by Semon and Wolff in 1933, who carried out premortem and postmortem examination of the eyes of a patient with severe SLE, and demonstrated an isolated break in Bruch’s membrane with overlying retinal pigment epithelium (RPE) and neurosensory retinal detachments [13].

Lupus choroidopathy may occur either independently or in association with lupus retinopathy, which is referred to as lupus chorioretinopathy. Lupus chorioretinopathy is characterized by serous elevations of RPE and/or the sensory retina. The unilateral or bilateral blurring of vision is the most common presenting symptom. Severe visual loss possibly suggests macular involvement. The choroidal effusion may result in secondary angle-closure glaucoma with the subsequent development of intraocular hypertension. Fundoscopy may be able to identify significant serous retinal detachments; however, SD-OCT may detect serous retinal detachments even at very early stages and is a valuable tool in measuring the changes in subretinal and intraretinal fluid over time. Fluorescein angiography may demonstrate multiple areas of leakage through RPE with the accumulation of the dye into the subretinal space. Exudative lesions have also been reported. Indocyanine green (ICG) angiography can also be used to identify areas of leakage in active choroidal disease. Histopathologic findings may include marked choroidal inflammation with diffuse lymphocytic infiltration and deposition of immunoglobulin and complement into the choroidal blood vessels along with choroidal vasculitis and RPE damage [14,15].

The pathogenesis of lupus chorioretinopathy remains unclear; however, it is generally agreed upon that increased permeability of the choroidal and retinal vessels, secondary to either elevated hydrostatic pressure or ischemia, is a prerequisite for this condition. As a result of the systemic immune processes and immune-complex deposition in the basement membrane of choroidal vessels, immune-mediated choroidal vascular compromise may occur. This leads to ischemia of the overlying RPE cells, which subsequently leads to a breakdown of the normally impermeable blood-retinal barrier provided by this layer. This allows the passage of leaked choroidal fluid across RPE, thereby producing elevations of the neurosensory retina [12]. However, some investigators believe that RPE dysfunction, independent of the underlying choroidal vascular compromise, may play a role in the development of chorioretinopathy [16].

Our patient was a known case of SLE and presented with decreased vision and bilateral serous retinal detachment. Based on these findings, our differential diagnoses included bilateral, diffuse, immune-mediated conditions, such as lupus chorioretinopathy, Vogt-Koyanagi-Harada (VKH) syndrome, and sympathetic ophthalmia. VKH syndrome is an autoimmune granulomatous disease that may also be associated with multifocal serous retinal detachment and choroidal swelling. However, patients with this condition also have non-ocular findings, such as meningismus, tinnitus, vertigo, vitiligo, and alopecia [17]. The absence of these findings ruled out VKH syndrome in our patient. Multifocal neurosensory retinal detachment is also seen in sympathetic ophthalmia; however, this condition was excluded as our patient lacked a history of any surgical or penetrating trauma to the eye, which is often the inciting event in the development of sympathetic ophthalmia [18]. Preeclampsia/eclampsia-associated retinopathy can also present with blurred vision, serous retinal detachment, choroidal dysfunction, retinal hemorrhages, cotton wool spots, and macular edema [19]. However, our patient was not pregnant. 

This case report highlights the significance of early recognition of lupus chorioretinopathy by differentiating it from other similar conditions, as it may be the sole indicator of active SLE in some cases. Patients should be monitored carefully, both by an ophthalmologist and a rheumatologist, since treatment of the underlying disease usually results in the resolution of the choroidopathy and subretinal fluid, as was seen in our patient. The established treatment for SLE includes high doses of steroids, steroid-sparing immunosuppressive medications, and biological agents [20].

## Conclusions

Chorioretinopathy secondary to SLE is a rarely described disease entity and the literature has a paucity of case reports of this condition. It is characterized by serous elevations of RPE and/or the sensory retina and may present with a unilateral or bilateral loss of vision. Lupus chorioretinopathy may sometimes indicate the presence of active SLE. Hence, early recognition, prompt referral, and multidisciplinary treatment strategies can play a key role in reducing the morbidity associated with this condition.
